# Dominant Red Coat Color in Holstein Cattle Is Associated with a Missense Mutation in the Coatomer Protein Complex, Subunit Alpha *(COPA)* Gene

**DOI:** 10.1371/journal.pone.0128969

**Published:** 2015-06-04

**Authors:** Ben Dorshorst, Corneliu Henegar, Xiaoping Liao, Markus Sällman Almén, Carl-Johan Rubin, Shosuke Ito, Kazumasa Wakamatsu, Paul Stothard, Brian Van Doormaal, Graham Plastow, Gregory S. Barsh, Leif Andersson

**Affiliations:** 1 Science for Life Laboratory, Department of Medical Biochemistry and Microbiology, Uppsala University, Uppsala, Sweden; 2 Department of Animal and Poultry Sciences, Virginia Tech, Blacksburg, Virginia, United States of America; 3 HudsonAlpha Institute for Biotechnology, Huntsville, Alabama, United States of America; 4 Livestock Gentec, Department of Agricultural, Food and Nutritional Science, University of Alberta, Edmonton, Alberta, Canada; 5 Department of Chemistry, Fujita Health University School of Health Sciences, Toyoake, Aichi, Japan; 6 Canadian Dairy Network, Guelph, Ontario, Canada; 7 Department of Genetics, Stanford University, Stanford, California, United States of America; 8 Science for Life Laboratory, Department of Animal Breeding and Genetics, Swedish University of Agricultural Sciences, Uppsala, Sweden; China Agricultural Univeristy, CHINA

## Abstract

Coat color in Holstein dairy cattle is primarily controlled by the *melanocortin 1 receptor* (*MC1R*) gene, a central determinant of black (eumelanin) vs. red/brown pheomelanin synthesis across animal species. The major *MC1R* alleles in Holsteins are Dominant *Black* (*MC1R^D^*) and *Recessive Red* (*MC1R^e^*). A novel form of dominant red coat color was first observed in an animal born in 1980. The mutation underlying this phenotype was named *Dominant Red* and is epistatic to the constitutively activated *MC1R^D^*. Here we show that a missense mutation in the *coatomer protein complex*, *subunit alpha* (*COPA*), a gene with previously no known role in pigmentation synthesis, is completely associated with Dominant Red in Holstein dairy cattle. The mutation results in an arginine to cysteine substitution at an amino acid residue completely conserved across eukaryotes. Despite this high level of conservation we show that both heterozygotes and homozygotes are healthy and viable. Analysis of hair pigment composition shows that the Dominant Red phenotype is similar to the *MC1R* Recessive Red phenotype, although less effective at reducing eumelanin synthesis. RNA-seq data similarly show that Dominant Red animals achieve predominantly pheomelanin synthesis by downregulating genes normally required for eumelanin synthesis. COPA is a component of the coat protein I seven subunit complex that is involved with retrograde and cis-Golgi intracellular coated vesicle transport of both protein and RNA cargo. This suggests that Dominant Red may be caused by aberrant MC1R protein or mRNA trafficking within the highly compartmentalized melanocyte, mimicking the effect of the Recessive Red loss of function *MC1R* allele.

## Introduction

The vast majority of dairy cattle of the Holstein breed display a black and white spotted coat color while a subset of this breed are red and white spotted. The *melanocortin 1 receptor* (*MC1R*) gene determines the basic coat color in cattle and in Holsteins there are four known *MC1R* alleles: Dominant Black (*MC1R* ^*D*^), Black/Red (*MC1R* ^*BR*^), the ancestral wild-type allele (*MC1R* ^*+*^) and Recessive Red (*MC1R* ^*e*^) [1–3]. The order of dominance is *MC1R* ^*D*^>*MC1R* ^*BR*^>*MC1R* ^*+*^>*MC1R* ^*e*^. Holsteins homozygous or heterozygous for any combination of the *MC1R* ^*+*^ or *MC1R* ^*e*^ allele are red, while animals heterozygous or homozygous for *MC1R* ^*D*^ are black. Black/Red animals are born red and change to black typically between two to six months of age. The causal mutations for *MC1R* ^*D*^ and *MC1R* ^*e*^ have been identified as constitutive activation and loss of function of *MC1R*, respectively [1,2]. *MC1R* ^*BR*^ has recently been shown to be a fourth *MC1R* allele through haplotype and linkage analysis with the causal mutation yet unidentified [3]. In Holsteins, *MC1R* ^*BR*^ and *MC1R* ^*+*^ are both rare, such that red vs. black coat color is primarily determined by the segregation of *MC1R* ^*D*^ and *MC1R* ^*e*^. Holstein breeders have become accustomed to the concept of red coat color being recessive to black and extensively utilize genetic testing of these *MC1R* mutations to mate carriers of *MC1R* ^*e*^ and/or *MC1R* ^*+*^ together to produce red offspring.

**Fig 1:**
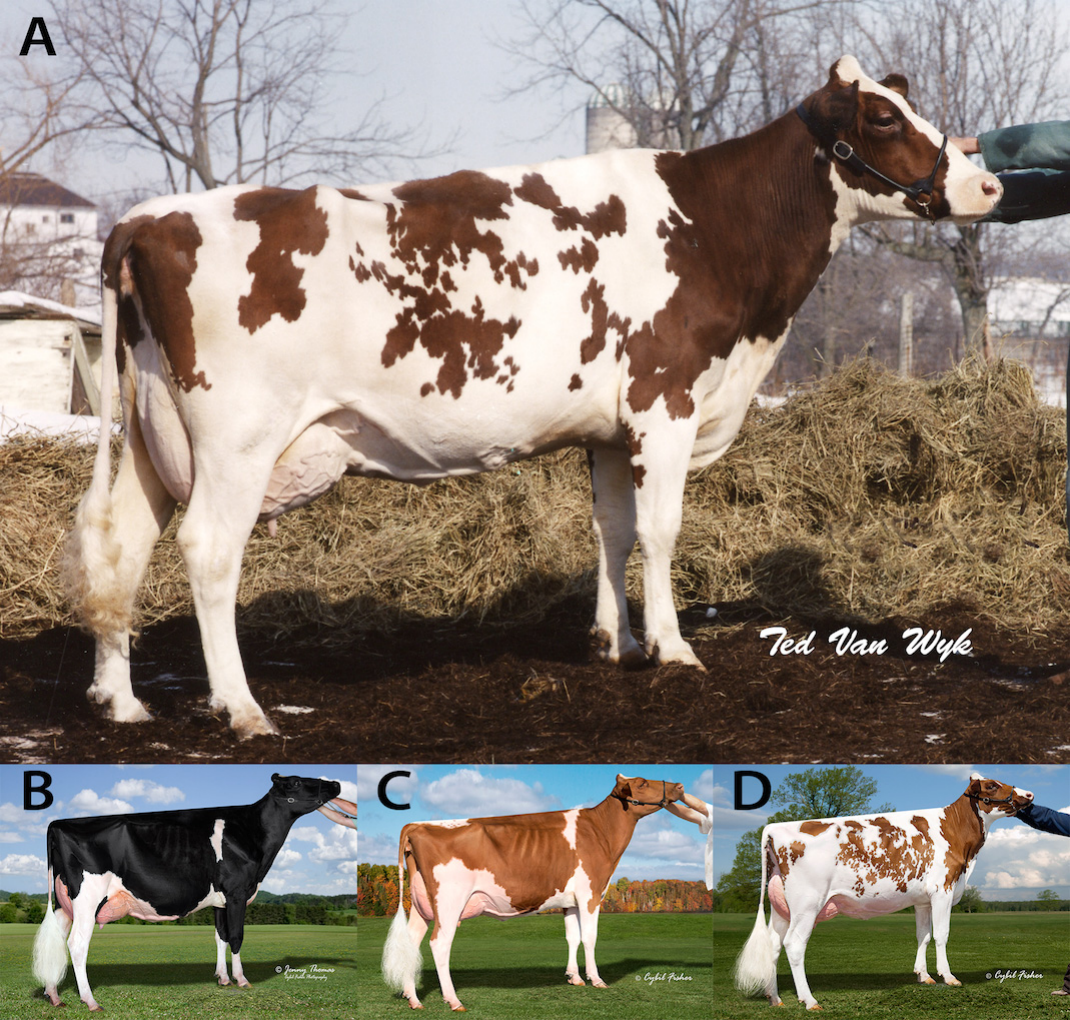
Dominant Red (*DR* ^*DR*^ */DR* ^*+*^, MC1R^*D*^ */-*), Dominant Black (*DR* ^*+*^ */DR* ^*+*^, MC1R^*D*^ */-*) and Recessive Red (*DR* ^*+*^ */DR* ^*+*^, MC1R^*e*^ */MC1R* ^*e*^) phenotypes. (A) The first animal to display the Dominant Red phenotype was SURINAM SHEIK ROSABEL-RED (HOCANF3541221), born in 1980 in Ontario Canada. (B) A Dominant Black Holstein female. (C) A Recessive Red Holstein female. (D) A Dominant Red Holstein female. Reprinted under a CC BY license, with permission from (A) Canadian Livestock Photography Inc., original copyright 1985 and (B, C and D) Cybil Fisher Photography, original copyright 2014.

In 1980, a female Holstein calf (HOCANF3541221, SURINAM SHEIK ROSABEL-RED) was born in Canada that displayed the typical red Holstein coat color phenotype (Fig 1), but from parents that were not thought to carry either of the *MC1R* alleles associated with red color based on pedigree. Genetic testing verified that this red animal was homozygous for *MC1R* ^*D*^ but yet approximately 50% of her progeny were phenotypically red, suggesting the presence of a new dominant form of red coat color, which was termed “Variant Red” [4]. At the time, this name reflected the unknown origin and mode of inheritance of this new genetic cause of red coat color. Here, we refer to this form of red coat color as “Dominant Red” (DR) to more clearly describe the mode of inheritance and differentiate it from the Recessive Red form of coat color caused by *MC1R*. We propose that the *Dominant Red* locus is designated *DR* with two alleles, the derivative allele *DR* ^*DR*^ and the ancestral or wild-type allele *DR* ^*+*^. The ability of *DR* ^*DR*^ to override the production of black pigment caused by a constitutively activated MC1R (*MC1R* ^*D*^), the central mechanism of pigment type switching in mammals, indicates that the DR phenotype represents a valuable opportunity to study novel aspects of pigmentation biology.

In a previous study the *beta-defensin 103* (*DEFB103*) gene on *Bos taurus* autosome (BTA) 27 was suggested as being associated with DR [5]. However, this analysis was limited to five candidate genes with known roles in pigmentation biology and found only marginal evidence for genetic linkage (LOD = 3.26). Here, we use whole genome linkage and association mapping techniques coupled with whole genome sequencing to show that in fact *DR* is located on BTA3 and is completely associated with a single missense mutation in the *coatomer protein complex*, *subunit alpha* (*COPA*) gene.

## Results

### Hair Pigment Analysis

Studies of coat color in laboratory mice indicate that the effect of MC1R signaling on hair color represents a switch between the synthesis of two alternative pigment types, red/yellow pheomelanin (caused by loss-of-function *MC1R* mutations) vs. black/brown eumelanin (caused by mutations that constitutively activate MC1R).

Hair samples collected from Dominant Black (*DR* ^*+*^/*DR* ^*+*^, *MC1R* ^*D*^/*-*), Dominant Red (*DR* ^*DR*^/*DR* ^*+*^, *MC1R* ^*D*^/*MC1R* ^*D*^) and Recessive Red (*DR* ^*+*^/*DR* ^*+*^; *MC1R* ^*e*^/*MC1R* ^*e*^) animals were analyzed for pigment content and composition [6]. Absorbance of solubilized hair pigment at 500 nm (Soluene-350, A500/mg) is an indicator of total melanin [7]. By this criterion, samples from Dominant Black animals had the highest melanin levels, Dominant Red samples were intermediate, and Recessive Red samples had the lowest levels (Table 1). We used HPLC analysis of melanin degradation products to measure individual pigment types. Pheomelanin content as assessed by 4-amino-3-hydroxyphenylalanine (4-AHP) or thiazole-2,4,5-tricarboxylic acid (TTCA) was approximately 50- to 60-fold and 12-fold higher respectively in Dominant Red and Recessive Red hair samples as compared to Dominant Black [8,9]. Eumelanin content, as assessed by pyrrole-2,3,5-tricarboxylic acid (PTCA) content [8], was highest in Dominant Black hair samples; Dominant Red samples exhibited PTCA levels that were approximately 8-fold lower, and Recessive Red levels were 23-fold lower (Table 1). Although the difference between Dominant Red and Recessive Red PTCA levels (approximately 3-fold) did not achieve statistical significance (*P* = 0.09), when the ratios of 4-AHP/PTCA or TTCA/PTCA were compared between Dominant Red and Recessive Red, the differences were significant (*P*<0.001). The Soluene-350 A650/A500 ratio, with higher values representing black/brown color and lower values representing yellow/red color were also significantly different for all three groups with Dominant Black samples having the highest value and Dominant Red being intermediate.

**Table 1:** Hair pigment content and composition.

	Dominant Black^ ^	Dominant Red^ ^	Recessive Red^ ^
Assay	*n = 5* ^* *^	*n = 8* ^* *^	*n = 9* ^ ^
Soluene-350 (A500/mg)	0.549^A^	0.247^B^	0.173^C^
	*0*.*027*	*0*.*021*	*0*.*020*
PTCA (ng/mg)	1918.0^A^	246.0^B^	82.8^B^
	*85*.*1*	*67*.*3*	*63*.*4*
4-AHP (ng/mg)	32.8^A^	1943.3^B^	1610.8^B^
	*227*.*0*	*179*.*5*	*169*.*2*
TTCA (ng/mg)	49.9^A^	623.9^B^	587.6^B^
	*37*.*6*	*29*.*7*	*28*.*0*
4-AHP/PTCA	0.0^A^	9.2^B^	19.6^C^
	*1*.*5*	*1*.*2*	*1*.*1*
TTCA/PTCA	0.0^A^	3.3^B^	7.7^C^
	*1*.*0*	*0*.*8*	*0*.*7*
Soluene-350 A650/A500	0.275^A^	0.159^B^	0.132^C^
	*0*.*008* ^ ^	*0*.*007* ^ ^	*0*.*006* ^ ^

Standard error in italics below respective mean.

^A B C^ Means with significant difference (*P*<0.05) indicated by different superscript letters.

These results indicate that similarities in hair color phenotype between Dominant Red and Recessive Red reflect similarities in pigment type synthesis, namely, production of pheomelanin rather than eumelanin, although suppression of eumelanin synthesis by Dominant Red is not as effective as in Recessive Red.

### Genetic Mapping

**Fig 2:**
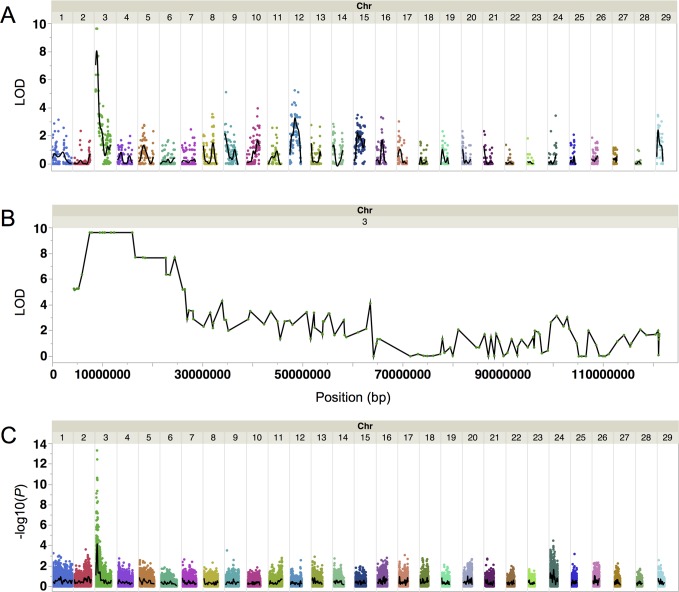
Linkage and genome-wide association mapping of Dominant Red. (A) Whole genome linkage mapping using a single half-sib family of 15 DR and 17 WT animals shows Dominant Red is located on BTA3. The black line is a cubic spline smoother with a lambda of 0.05. (B) Linkage mapping shows eight contiguous SNPs on BTA3 in complete association with Dominant Red across a 10.7 Mb region. (C) Genome-wide association analysis in 95 DR and more than 500 WT animals identifies a significant association on BTA3 within the 10.7 Mb region identified by linkage mapping. The most significant marker is ARS-BFGL-NGS-94819 @ 9,722,400 bp. The black line is a cubic spline smoother with a lambda of 0.05.

Genetic mapping of *DR* was performed using a heterozygous DR bull (HOCANM9626808, MORSAN RED GOLD) as well as 15 DR and 17 non-DR progeny, including their dams, comprising a single half-sib family. Two point linkage analysis between coat color and 2,752 autosomal markers, which were heterozygous in the DR sire, was performed using CRI-MAP 2.503 [10]. The highest genome-wide LOD score of 9.6 was found on BTA3 (Fig 2A). Eight contiguous SNPs on BTA3 had this same LOD score and showed complete linkage with the DR phenotype in this half-sib family (Fig 2B). The nearest flanking markers showing recombination to *DR* define a 10.7 Mb region containing the *DR* causal variant by linkage analysis, from BTA-21472-no-rs @ 5,886,143 bp to ARS-BFGL-NGS-20167 @ 16,557,950 bp. The UMD3.1/bosTau6 genome assembly was used for all analyses [11].

A genome-wide association analysis (GWAS) was performed using 95 DR heterozygous animals, 55 non-DR animals closely related to the 95 DR individuals and 500 non-DR animals randomly selected from the population. The 500 non-DR randomly selected animals had previously been genotyped for genomic selection purposes. The DFAM procedure of the PLINK [12] software package was used to perform a family-based association test for disease traits, a method which accommodates the use of unrelated individuals and case/control traits. A single peak on BTA3 was identified as being most highly associated with the DR phenotype (Fig 2C). A single SNP (ARS-BFGL-NGS-94819 @ 9,722,400 bp) showed the strongest genome-wide association of-log10(*P*) = 13.3 with all 95 DR animals having at least one copy of the DR associated allele. However, the DR associated allele of ARS-BFGL-NGS-94819 had a frequency of 6.7% in the 500 non-DR animals, indicating that it would result in a high false positive rate if this SNP were used as a genetic marker for DR.

We searched for a haplotype that was identical by descent (IBD) in DR animals within the 10.7 Mb region defined by linkage mapping and confirmed by GWAS results in a larger group of animals. The major allele at each SNP across this region in 95 DR animals was considered to represent the allele present on the putative IBD haplotype. The minimal IBD haplotype was defined as the region where all of the 95 DR animals had at least one copy of the major allele. A region satisfying this criterion was identified from ARS-BFGL-NGS-112616 @ 7,906,099 bp to ARS-BFGL-NGS-1429 @ 10,462,387 bp, spanning 2.6 Mb of BTA3 (S1 Fig). A single individual did not conform to this rule, possibly due to recombination events that we could not resolve. When possible, more than one animal in discordance with this definition was used to define the boundary of the IBD haplotype.

### Whole Genome Sequencing

A heterozygous DR individual (HOCANF9845383, BROEDERDALE MALTBY CAMILLA) was selected for whole genome sequencing in order to identify all candidate causal sequence variants within the 2.6 Mb IBD region. This individual was selected based on maximum homozygosity across the IBD region (S1 Fig, top row) in order to minimize the number of non-DR associated variants. This means that we selected an animal whose *DR* ^*+*^ chromosome was closely related to its *DR* ^*DR*^ chromosome. At the time of sequencing no *DR* ^*DR*^ */DR* ^*DR*^ homozygotes were yet known. From the whole genome sequencing data 8,485 SNPs were detected within the IBD region, 77 of which cause an amino acid change and 51 of which were heterozygous. Sequence data from the 1000 Bull Genomes Project [13] was used to exclude variants that have been detected in non-DR animals, which further narrowed the list of candidate causal SNPs to 25. Variant coverage, quality scores, and manual inspection of the aligned reads were used to evaluate which SNPs had the most evidence of being true variants. SNP calling criteria was purposefully set at a low threshold to minimize exclusion of true variants.

The six candidate causal SNPs with the highest evidence of being true variants were genotyped in a panel of 20 DR heterozygous animals (S1 Table). Three of the SNPs were excluded as false positives as they were not detected by conventional sequencing methods in the same animal used for whole genome sequencing. This suggests that all other remaining candidate causal SNPs with lower quality scores were also likely to be false positives. Two of the SNPs were excluded due to homozygotes of alternative alleles being found in the panel of 20 DR heterozygotes. The one remaining SNP (C>T @ BTA3:9,479,761 bp) had the highest quality score out of the 25 candidate causal SNPs detected within the entire IBD region and was found to be heterozygous in all 20 DR heterozygotes in the panel (S1 Table). No insertions or deletions that were also heterozygous were detected in the IBD region.

### Analysis of Structural Variants in the IBD Region

To determine if a structural variant is associated with the Dominant Red phenotype read depth and deviant mate-pairs within the IBD region were analyzed for insert size, read order and strand orientation. The sequence from the Dominant Red individual was compared to a Dominant Black Holstein reference individual (HOCANM10705608, BRAEDALE GOLDWYN) that had been sequenced to the same average depth. Normalized read depth in windows was analyzed first within both individuals separately using CNVnator and then between individuals, using CNV-seq. The result of the SV analysis is summarized in S2 Fig. Using CNVnator, two regions (BTA3:8,508,750–8,539,500 and BTA3:8,611,500–8,679,000) were called as deletions in both individuals and likely represent assembly/mapping artifacts. In addition, one region (BTA3:8,731,500–8,752,500) was called as a deletion in the reference individual whereas the Dominant Red individual displayed a normal read depth. However, this region does not have support from the CNV-seq analysis or any deviant reads and is likely a false positive. No regions were found to significantly deviate in read depth among individuals using CNV-seq. Analysis of deviant mate-pairs with SVDetect found one putative duplication in Dominant Red (BTA3:9,007,145–9,008,020). This duplication is also supported in the reference individual and may be an assembly artifact. Taken together, no likely candidate causative structural variants within the IBD region specific to the DR individual were identified.

### Causal Variant Validation and Conservation

Due to the lack of structural variants and insertions or deletions associated with DR in the IBD region the C>T SNP at BTA3:9,479,761 bp was considered to be the most likely DR candidate causal variant. A diagnostic test for this SNP was developed and used to genotype 78 known DR heterozygotes. All 78 animals were found to be heterozygous at this SNP. Of 189 randomly selected non-DR animals and 94 non-DR relatives of DR animals, all were homozygous for the wild-type allele. These results demonstrate the complete association of the DR candidate causal variant with DR phenotype.

**Fig 3:**
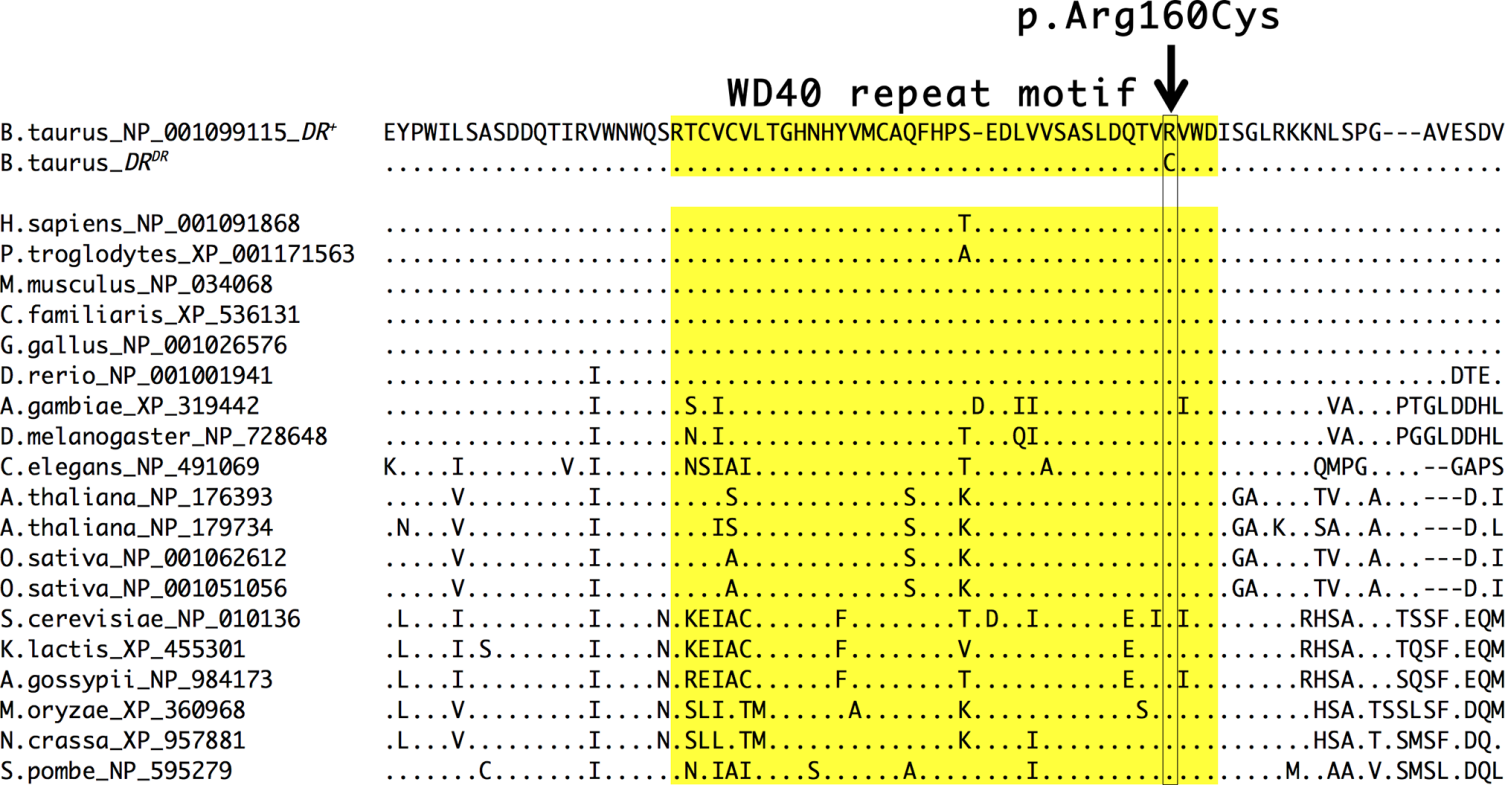
Evolutionary conservation of Dominant Red associated COPA mutation. Multiple sequence alignment using MUSCLE [16] of COPA and orthologues indicates high conservation across 17 species from mammals to yeast. The arginine (R) to cysteine (C) amino acid substitution caused by the Dominant Red SNP is indicated with the arrow and is completely conserved in all species with available sequences. The WD40 repeat motif is highlighted in yellow.

The candidate causal variant at BTA3:9,479,761 bp is located in the coding region of *COPA*, a gene not previously known to have a direct role in pigmentation biology. In the COPA mRNA transcript NM_001105645 this SNP is located at coding position 478 and results in an arginine to cysteine substitution at amino acid position 160 in NP_001099115 (c.478C>T p.Arg160Cys). Protein sequence alignment to all COPA orthologs present in NCBI databases reveal that an arginine amino acid is completely conserved at this position from mammals to yeast, residing in a highly conserved WD40 repeat motif (Fig 3). Sequence conservation across such a broad range of species suggests evolutionary constraint due to an important function; indeed, the Arg160Cys substitution in human COPA is predicted to be probably damaging or damaging by PolyPhen-2 [14] and SIFT [15], respectively. The DR candidate causal variant is therefore likely to have a significant effect on the function of this conserved region.

Due to the high evolutionary conservation of the amino acid residue affected by the DR candidate causal variant and the fact that no homozygotes for DR had previously been documented, we considered the possibility that the DR variant may be detrimental in the homozygous state. The DR diagnostic test was used to investigate this scenario by genotyping red individuals that were progeny of two DR parents. Out of 14 animals tested to date four individuals were determined to be homozygous for the DR candidate causal variant. All animals appeared healthy indicating that DR homozygotes are viable.

### RNA-Seq Analysis of Dominant Red Skin

**Fig 4:**
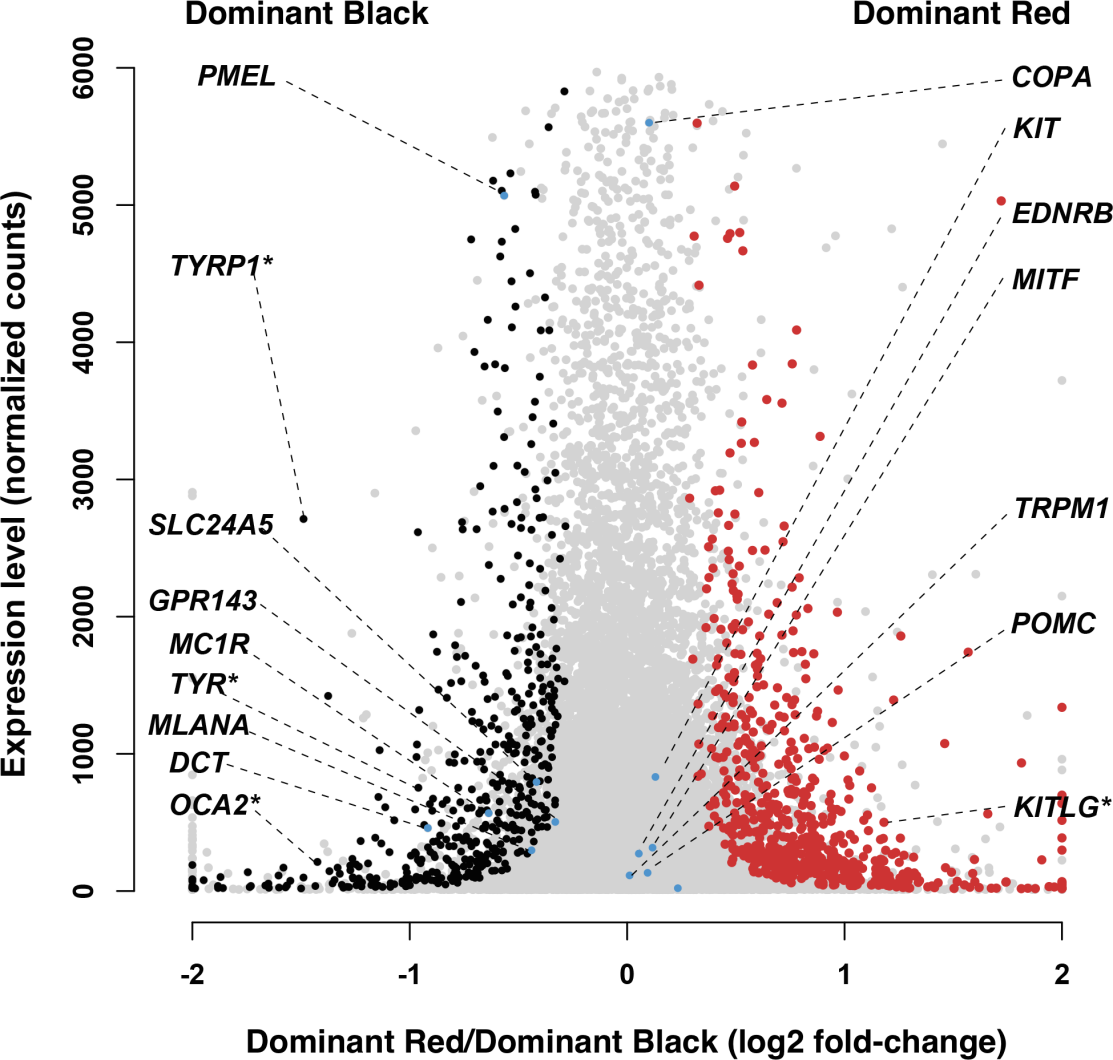
Differential expression results comparing Dominant Red (*DR* ^*DR*^ */DR* ^*+*^, *MC1R* ^*D*^ */MC1R* ^*D*^) and Dominant Black (*DR* ^*+*^ */DR* ^*+*^, *MC1R* ^*D*^ */-*) skin samples. Gene expression levels (normalized gene counts averaged across the six analyzed samples) are represented as a function of log2-fold changes of Dominant Red versus Dominant Black samples. Genes that are significantly overexpressed in Dominant Black skin samples are depicted in black, those overexpressed in Dominant Red samples are illustrated in red, while non-significant changes are illustrated in gray. Genes previously implicated in melanocyte biology and/or pigment type-switching are annotated (selected genes that do not exhibit a significant difference at FDR<0.1 are highlighted in blue while the ones showing significant expression changes are marked additionally with an asterisk associated to the gene symbol).

To gain additional insight into the mechanisms underlying *Dominant Red* gene action, we compared gene expression profiles between skin from Dominant Red (*DR* ^*DR*^/*DR* ^*+*^, *MC1R* ^*D*^/*MC1R* ^*D*^) and Dominant Black (*DR* ^*+*^/*DR* ^*+*^, *MC1R* ^*D*^/*-*) animals. Skin biopsies from three individuals of each genotype were used as the source of cDNA libraries that were sequenced on an Illumina HiSeq instrument and then aligned against the bovine reference genome (UMD3.1/bosTau6). Differentially expressed genes were identified with DESeq2 [17]; among 15,143 genes that were assayed, 111 and 1104 exhibited significantly different levels of expression at a FDR<0.05 and a FDR<0.1, respectively (Fig 4, S2 Table).

Fig 4 depicts those results with the level of gene expression plotted as a function of the log2 ratio of Dominant Red/Dominant Black; thus, genes overexpressed in Dominant Black compared to Dominant Red skin are on the left side of the plot, and vice versa, with genes exhibiting a significant difference (FDR<0.1) highlighted in black or red, respectively. Genes previously implicated in melanocyte biology and/or pigment type-switching are annotated (selected genes that do not exhibit a significant difference at FDR<0.1 are highlighted in blue). Notably, *TYRP1*, *TYR*, and *OCA2*, which encode proteins previously known to be required for and upregulated during eumelanogenesis, are overexpressed in Dominant Black compared to Dominant Red skin. Two additional genes (*PMEL* and *DCT*), also required for and upregulated during eumelanogenesis, are also overexpressed in Dominant Black compared to Dominant Red skin but at an FDR>0.1. We also considered the three major paracrine ligand-receptor systems involved in melanocyte biology and pigment type-switching: *ASIP* and *POMC—MC1R*, *KITLG—KIT*, *and EDN3—ENDRB*; these genes were either not detected or not differentially expressed, with the exception of *KITLG*, which is overexpressed in Dominant Red compared to Dominant Black skin. Taken together, these results suggest that the biochemical mechanism of pheomelanin production in Dominant Red skin is identical to that caused by MC1R deficiency, and that the phenotype arises due to melanocyte-autonomous alterations in gene expression.

We also carried out a functional annotation of differentially expressed genes using the FunNet tool [18]. The most striking result, apparent from a KEGG analysis is that the four pathways most overrepresented in differentially expressed genes are Endocytosis, MAPK signaling, ER protein processing, and Antigen processing and presentation, all of which are increased in Dominant Red compared to Dominant Black skin (Table 2). These observations are consistent with impairment of protein trafficking due to the *COPA* missense alteration, and upregulation of compensatory pathways.

**Table 2:** KEGG pathways significantly enriched in genes that are overexpressed in Dominant Red skin samples.

KEGG Pathway	Enrichment *P*-value
Endocytosis	1.65x10^-3^
Protein processing in endoplasmic reticulum	2.19x10^-2^
Antigen processing and presentation	2.01x10^-2^
Phosphatidylinositol signaling system	2.71x10^-2^
Natural killer cell mediated cytotoxicity	5.39x10^-3^
Jak-STAT signaling pathway	4.45x10^-2^
Oocyte meiosis	1.75x10^-2^
Lysine degradation	3.15x10^-2^

### Origin of the Dominant Red Mutation

The *DR* ^*DR*^ mutation must have occurred on the IBD haplotype described earlier in the germline of either the sire or dam of the DR founder animal ROSABEL. Whole genome genotyping data was available on some of the ancestors of ROSABEL: A PUGET-SOUND SHEIK (sire), PROVIN MTN IVANHOE JEWEL (paternal grand sire), OSBORNDALE IVANHOE (paternal great grand sire) and SEILING ROCKMAN (maternal great grand sire). Using the IBD haplotype data we were able to exclude the sire of ROSABEL as the germline source of the *DR* variant as this individual did not carry the *DR* IBD haplotype. This indicates that the *DR* mutation occurred in either the germline of ROSABEL’s dam or very early in embryonic development of ROSABEL herself. A DNA sample from ROSABEL’s dam was not available, however the maternal great grand sire had previously been genotyped and was found to carry the *DR* IBD haplotype. This individual (HOCANM275932, SEILING ROCKMAN) had at least one copy of the major allele among the 95 Dominant Red heterozygotes at each locus over an expanded 18 Mb region (S1 Fig). This individual would be expected to be wild-type for the DR associated mutation in COPA since he was phenotypically black, although a sample was not available for confirmation. Further genotyping of ROSABEL’s maternal ancestors would be needed to conclusively determine which individual contributed the haplotype on which the *DR* variant originated. However, due to the size of the region concordant between SEILING ROCKMAN and the major allele in 95 DR heterozygotes it is likely that this individual contributed the chromosome on which the *DR* ^*DR*^ mutation later occurred.

A bull thought to be unrelated to ROSABEL (HOUSAM2141664, MAPLE-VANE SURPRISE-RED) has been reported to transmit a similar dominant red phenotype. A semen sample of this bull was obtained from the USDA National Animal Germplasm Program and genotyped for the *COPA* mutation using the diagnostic test. This individual was in fact heterozygous for the DR-associated missense mutation. Haplotype analysis found that SURPRISE was concordant for the DR associated allele at 19 out of 21 SNPs in the IBD region for which data was available. The same mutation may have occurred independently; alternatively, it is possible that there is an error in the pedigree of SURPRISE, and that the DR-associated allele in SURPRISE originated in ROSABEL.

## Discussion

Here we provide genetic evidence that a missense mutation in *COPA* causes Dominant Red, a novel dominant red coat color phenotype that recently evolved in the Holstein dairy cattle breed. The identification of the same region of BTA3 as being associated with DR using two different genetic mapping methods indicates that the previous results suggesting *DR* is located on BTA27 and possibly caused by a mutation in *DEFB103* are incorrect [5]. Haplotype analysis narrowed the region containing the DR causal variant to a 2.6 Mb IBD region of BTA3. The sire of the DR founder animal did not carry this IBD haplotype indicating that the causal mutation originated in the maternal germline of the dam or early during embryonic development of the DR founder, ROSABEL. Whole genome sequencing identified six candidate causal variants in the IBD region, five of which were excluded after screening a panel of 20 known DR heterozygotes. The one remaining SNP at BTA3:9,479,761 bp was concordant with expected *DR* genotype in a panel of 78 DR and 283 non-DR animals. This SNP results in an arginine to cysteine substitution at amino acid 160 of COPA, predicted to impair COPA function, and is consistent with differential gene expression detected using RNA-Seq analysis of Dominant Red compared to Dominant Black skin. Taken together with additional results from hair biochemical and RNA-Seq analyses, our results provide strong evidence that a reduction of COPA activity causes a red coat color by interfering with the effects of melanocortin receptor signaling.

*COPA* encodes the α-COP subunit of the coat protein I (COPI) seven subunit complex that is involved with intracellular coated vesicle transport. Originally characterized as a critical component for retrograde transport of luminal and membrane proteins from the Golgi to the endoplasmic reticulum and between Golgi compartments [19–21], COPI participates in a range of macromolecular complexes with diverse cargoes, including ribonucleoproteins that modulate trafficking of RNA in neuronal cells [22,23]. Because the pattern of mRNA expression in Dominant Red skin mimics that observed in Recessive Red skin, we speculate that reduced COPA activity caused by the Arg160Cys substitution impairs MC1R signaling via altered localization of its mRNA and/or protein. Like neurons, melanocytes are highly compartmentalized cells, and it is possible that *MC1R* mRNA is normally transported to specific locations in dendrites, a process disrupted by the DR mutation. Alternatively, or in addition, the DR mutation may interfere with MC1R receptor recycling between plasma membrane and endosomal compartments (reviewed in [24]).

Previous studies of COPA function have focused mostly on cell biology and biochemical approaches; indeed, heritable variation of *COPA* has not been described previously in mammals, and Dominant Red cattle represent a unique opportunity to study COPI function in an organismal context. Loss-of-function for *COPA*, *COPB*, or *COPB2* in zebrafish causes embryonic lethality [24], and it therefore seems likely that the Arg160Cys mutation is hypomorphic. An analogous argument has been made for a missense mutation of *Archain 1* (*Arcn1*) in laboratory mice, which causes neurodegeneration and pigmentary dilution; *Arcn1* encodes the delta-subunit of COPI [23]. Pigmentary dilution in the mouse mutant was suggested to involve defects in trafficking of proteins required for melanogenesis; our results suggest the additional possibility that *Arcn1*-related pigmentary changes represent a reduction of MC1R signaling, an idea that could be tested by examination of *Arcn1*; *Mc1r* double mutants.

In cattle, both Recessive Red and Dominant Red animals display variation in the intensity of red coloration over the entire body as well as a tendency to darken in the extremities, possibly due to modifier loci or non-genetic effects. However, some have suggested that Dominant Red animals are more often a darker shade of red than Recessive Red animals, consistent with our observation that eumelanin is reduced more in Recessive Red than in Dominant Red.

Visually we observed that Dominant Red animals had a tendency to have darker hairs intermixed with lighter hairs while Recessive Red animals displayed a more uniform intra-hair pigmentation color. However, we observed variation in shade of red within both groups of red animals. We also observed that, at birth, Dominant Red animals were indistinguishable from Recessive Red and that any darkening that might occur in some Dominant Red animals typically takes place after four to six months of age. These observations on color are all based on Dominant Red heterozygotes since very few homozygotes are in existence due to the recent occurrence of this mutation. The work reported here provides the basis for careful evaluation of potential additive and epistatic relationships between Dominant Red and Recessive Red in livestock, an opportunity that is usually limited to model organisms.

During the preparation of this manuscript it has come to our attention that another group has independently identified the very same COPA mutation as associated with Dominant Red color in Holsteins, providing further support that this missense mutation is causal (Bourneuf E, Otz E, Michot P, Grohs P, Piton C, Deloche M.-C., Cornier M, Delahaye M, Fritz S, Leclerc H, Longin C, Boukadiri A, Saintilan R, Créchet F, Mosca M, Guillaume F, Bouet S, Baur A, Vasilescu A, Genestout L, Allais-Bonnet A, Rocha D, Colle M.-A., Klopp C, Esquerré D, Barbey S, Fayolle G, Danchin-Burge C, Bed'Hom B, Daetwyler H D, Boichard D, Pin D and Capitan A, in preparation).

## Conclusions

Here we show that a mutation causing an arginine to cysteine substitution at a highly conserved position in the fourth WD40 repeat motif of COPA causes the striking Dominant Red phenotype in Holstein cattle. The mutation affects a key component of the fundamental cellular process of coated vesicle transport with no known adverse effects on other traits. Hair chemical analyses indicate a shift from eumelanin to pheomelanin production in Dominant Red individuals, similar to what is seen in the Recessive Red phenotype caused by a loss-of-function mutation in *MC1R*. RNA-Seq analyses indicate that Dominant Red skin samples have decreased expression of genes necessary for eumelanin synthesis (*TYRP1*, *TYR*, and *OCA2*). Expression of these genes is upregulated by MC1R signaling, which suggests that altered COPA activity in Dominant Red skin samples impairs eumelanin synthesis by selectively inhibiting MC1R signaling. The COPA mutation associated with Dominant Red provides an opportunity to study the potential relationship between coated vesicle transport and MC1R signaling.

## Materials and Methods

### Genetic nomenclature

In cattle as well as in many other domestic animals, variation at *MC1R* was originally recognized and described as the “Extension” (*E*) locus. In accord with the HUGO Gene Nomenclature Committee Recommendations [25], we use *MC1R* as the gene name instead of *E*, with superscripts *D*, *BR*, +, and *e* to refer to alleles. As described here, we provide very strong evidence that *Dominant Red* represents allelic variation in *COPA*; however, since there are no known *COPA* alleles in other animals yet, we refer to the ancestral and derivative *Dominant Red* alleles as *DR* ^*DR*^ and *DR* ^*+*^, respectively.

### Animals

Holstein cattle hair samples were obtained from dairy farmers in Canada and the USA unless stated otherwise. Where relevant and available, individuals are identified by their breed association registration number and name to facilitate lookup of additional information on breed association websites. Animals used for chemical hair pigment and RNA-Seq analyses were selected based on pedigree information so as to maximize within-group genotype similarity. Specifically, none of the animals used came from backgrounds that include *MC1R* ^*BR*^ or *MC1R* ^*+*^ (which are both rare in the Holstein breed), all Dominant Red and Dominant Black animals had no record of Recessive Red in their background, and all Recessive Red animals had no record of Dominant Red in their background. Additionally, all Dominant Red animals had one black and one red parent; thus, the phenotypic designations (and inferred genotypes) are: Dominant Black (*DR* ^*+*^/*DR* ^*+*^; *MC1R* ^*D*^/*MC1R* ^*D*^), Dominant Red (*DR* ^*DR*^/*DR* ^*+*^; *MC1R* ^*D*^/*MC1R* ^*D*^), and Recessive Red (*DR* ^*+*^/*DR* ^*+*^; *MC1R* ^*e*^/*MC1R* ^*e*^). This study utilized tissues and/or DNA obtained from domestic animals maintained by their owners. All samples were collected following best practice husbandry and veterinary procedures. No laboratory animals or laboratory animal research subject to IACUC regulations were used.

### Pigment Analysis

Hair was collected from the paralumbar fossa region of females approximately one year of age. Hair was cut immediately adjacent to the skin and wrapped in aluminum foil until analysis. Hair samples (15–20 mg) were homogenized with a Ten-Broeck homogenizer at a concentration of 10 mg/ml and 100 μl aliquots were subjected to Soluene-350 solubilization [7], alkaline hydrogen peroxide oxidation [8], and hydroiodic acid hydrolysis [9].

### Genotyping

The sire, progeny and respective dams of the half-sib family used for linkage analysis were genotyped on the BovineLD Genotyping BeadChip (Illumina) [26]. Animals used for the GWAS were genotyped on the BovineSNP50 Genotyping BeadChip (Illumina) [27] or genotypes were imputed to this level from a lower density SNP panel. Additional animals were targeted for genotyping through the established genomic selection system with assistance from Canadian Dairy Network, Holstein Association of Canada, and Holstein Association USA. Owners provided hair samples directly to commercial genotyping labs. A subset of samples was also sent by the owners to the researcher’s lab for follow up genotyping. These samples were composed of either hair or blood and were isolated using the Gentra Puregene kit (Qiagen). Animals that could be both Recessive Red and/or Dominant Red based on pedigree analysis were excluded from all analyses unless a negative genetic test for Recessive Red was obtained.

### Sequencing

SOLiD mate pair libraries were prepared from a Dominant Red individual (HOCANF9845383, BROEDERDALE MALTBY CAMILLA) according to the SOLiD System Mate-paired Library Preparation protocol of the Applied Biosystems SOLiD System (ABI). The libraries were sequenced on two flowchips using the SOLiD 5500xl platform. The initial libraries had a large number of duplicate reads so a paired-end library was then prepared and sequenced on the same machine. The resulting data was of good quality. The sequence data have been uploaded to the NCBI sequence read archive (SRA: SRS834811).

Short reads were aligned in color space against the UMD3.1/bosTau6 genome assembly using BWA v0.5.9 [28]. After mapping, the duplicates within each BAM file were marked using Picard v1.54 (http://broadinstitute.github.io/picard). Because the first two runs were using the same mate-pair library, the BAM files were merged and duplicates were marked again for removal.

Variants (SNPs and indels) were called using SAMtools v0.1.18 [29] mpileup with no quality filtering in order to not exclude any true positives with low quality scores. Genotypes from the 50K SNP chip were used to validate sequencing results. Genotypes were concordant between variant calling results and existing genotype data for all SNPs present in the IBD region that are also on the 50K SNP chip, 12 homozygous non-reference SNPs and 10 heterozygous SNPs.

SNPs and indels were annotated with predicted functional consequences using NGS-SNP [30]. Overlapping genes, transcripts, proteins, protein domains, and variants were included among the annotations, along with any known pathways or phenotypes linked to the genes in cattle or to their orthologues in humans. Sequence alignments were constructed between missense SNP-altered proteins their orthologues and scored using the SIFT [15] algorithm to predict the functional significance of protein substitutions. Variants were classified as “known” if the non-reference allele was present in the dbSNP database and “novel” otherwise. The source databases used by NGS-SNP during annotation include Ensembl release 68, dbSNP build 133, Entrez Gene, and UniProt release 2012_09.

### Detection of Structural Variations

In order to detect putative causative structural variations within the IBD region the two SOLiD mate-pair libraries for the Dominant Red individual was mapped against the UMD3.1/bosTau6 reference assembly. For comparison, two SOLiD mate-pair libraries from a Holstein bull (HOCANM10705608, BRAEDALE GOLDWYN) were downloaded from the NCBI sequence read archive (SRA: SRR592656, SRR592657) [31] and used as a reference since this individual does not have the Dominant Red phenotype. The reads from both individuals were mapped with MOSAIK v2.2 [32] using a hash size of 14 (-hs 14), allowing four mismatches per read (-mm 4), an alignment candidate threshold of 20 (-act 20) and otherwise default settings. Non-unique alignments were excluded and duplicated read pairs were removed with the Picard v1.123 (http://broadinstitute.github.io/picard) package’s MarkDuplicates utility before subsequent analyses.

Putative copy number variants (CNVs) were analyzed across BTA3 but only called within the IBD region. First the program CNV-seq [33] was used to compare read depth between the Dominant Red and reference individual in overlapping windows across the chromosome. Based on the default significance threshold of *P*<0.001 and log_2_-fold change threshold of ±0.6, CNV-seq automatically calculated a suitable window size of 2,979 bp. In order to call a significant CNV at least two consecutive windows needed to be significant. Second, the program CNVnator [34] was used to call putative CNVs based on read depth within both individuals separately in sliding windows of 750 bp across chromosome three. CNVnator combines nearby windows that display a similar CNV signal and calculates statistics for the resulting regions. A significance threshold of *P*<0.001 was used for the CNVnator analysis.

In addition to the read depth based analysis, the program SVDetect [35] was used to detect putative SVs based on deviant read mate-pairs. A read mate-pair is called deviant if it has an abnormal insert size, read order orientation or read strand orientation. This method makes it possible to detect copy number balanced variants such as inversions and translocations in addition to duplication and deletion events that affect read depth. SVDetect clusters reads into links that corresponds to a potential SV and classifies it based on how the mate-pairs deviates. Only high quality alignments were used by excluding reads with a mapping quality less than 20. For SVDetect, default settings were used to detect the abnormal mate-pairs. Deviant reads were detected and clustered into links separately for the Dominant Red and reference individual. The putative SVs were selected in the Dominant Red individual from links that were supported by at least five reads and had a final score of one (highest confidence). Any links crossing an assembly gap were removed. These candidates were then compared with the reference individual and overlapping links of the same type were deemed as assembly/alignment artifacts or not associated with the Dominant Red phenotype.

### Diagnostic Test

A diagnostic test was developed for the causal Dominant Red SNP (COPA c.478C>T p.Arg160Cys) at BTA3:9,479,761 bp using a Custom TaqMan SNP Genotyping Assays (ABI). Primers and probes were Forward: 5’-TCAGAAGACCTGGTCGTGTCA-3’, WT allele: 5’-VIC-CAAACGCGCACAGTC-NFQ-3’, DR allele: 5’-FAM-CCAAACGCACACAGTC-NFQ-3’ and Reverse: 5’-TGGGCAGCTCACCAGAAATATC-3’. Genotyping was performed according to the standard protocol using TaqMan Genotyping Master Mix (ABI).

### RNA-Seq

Punch biopsies were performed to obtain skin samples from six individuals (three Dominant Red, three Dominant Black), and then stored in RNALater at -80°C. Total RNA was isolated using RNeasy Fibrous Tissue Mini Kit (Qiagen), assessed for integrity with an Agilent Bioanalyzer instrument, and cDNA libraries then prepared with the TruSeq RNA Sample Preparation Kit v2 (Illumina). Individual libraries were multiplexed as six per lane and sequenced as paired-end 50 base pair reads on an Illumina HiSeq 2000 instrument at the Genome Sequencing Laboratory of the HudsonAlpha Institute.

RNA-Seq reads obtained for each sample were aligned against the bovine reference genome (UMD3.1/bosTau6) with TopHat2 software, using genomic sequence and transcript annotations obtained from Ensembl (release 72). On average ≈34 million paired-end reads were obtained for each sample, out of which ≈66% were mapped individually and ≈62% were mapped as concordant pairs against the reference transcriptome. Gene counts were computed from read alignments with GenomicRanges and GenomicFeatures packages from Bioconductor (release 2.14), and then used to test for differential expression between Dominant Red and Dominant Black skin samples with DESeq2 package from Bioconductor. DESeq2 relies on negative binomial generalized linear models to determine whether the number of counts for a transcript or gene is significantly different across a range of experimental conditions.

## Supporting Information

S1 Fig IBD haplotype for Dominant Red phenotype. A 2.6 Mb IBD haplotype in Dominant Red animals is located on BTA3 from ARS-BFGL-NGS-112616 @ 7,906,099 bp to ARS-BFGL-NGS-1429 @ 10,462,387 bp. Animals are in rows and SNPs are in columns. Phenotype is indicated in the first column with Dominant Red animals shaded in red and wild-type animals shaded in gray. SNPs are shown that span the entire 10.7 Mb region identified in the linkage analysis. Orange shading indicates homozygotes for the major allele, yellow shading indicates heterozygotes and blue shading indicates homozygotes for the minor allele. The IBD haplotype is shaded in gray and the SNP with the highest GWAS significance shaded in green (ARS-BFGL-NGS-94819 @ 9,722,400 bp). The four ancestors of the founder animal ROSABEL are indicated at the left of the figure. SEILING ROCKMAN shows a haplotype concordant with the major allele from Dominant Red animals extending beyond the 2.6 Mb IBD haplotype.
(TIFF)

S2 Fig Detection of structural variants within the IBD region. The IBD region was subject to analysis of candidate structural variations using whole genome sequencing reads from a Dominant Red (HOCANF9845383, BROEDERDALE MALTBY CAMILLA) and reference individual (HOCANM10705608, BRAEDALE GOLDWYN). The top track represents RefSeq Gene annotation. Normalized and GC corrected read depth (RD) for the two individuals are found in the bottom two tracks with the mean RD marked with a red line. Significant deletions from CNVnator (orange) and duplications from SVDetect (green) within each individual are overlaid on the RD graph. No other significant structural variations were detected, including analysis with CNV-seq to compare RD between individuals.
(TIFF)

S1 Table Dominant Red candidate causal mutation screening. Six SNPs identified by whole genome sequencing within the IBD region were screened on a panel of 20 DR animals. A “.” indicates the same genotype as the reference genome. Variant calling quality scores from whole genome sequencing analysis are listed in the top row of the table. Three SNPs (8,028,785 bp, 9,385,285 bp, and 9,943,108 bp) were found to be false positives from whole genome sequencing after Sanger sequencing using the same individual. Two SNPs (10,295,754 bp and 10,295,797 bp) were excluded as candidate causal mutations since not all DR individuals carried the variant allele. The remaining SNP (9,479,761 bp) was found to be heterozygous in all 20 DR individuals.
(XLSX)

S2 Table Results of differential expression analysis comparing Dominant Red (*DR* ^*DR*^ */DR* ^*+*^, *MC1R* ^*D*^ */MC1R* ^*D*^) and Dominant Black (*DR* ^*+*^ */DR* ^*+*^, *MC1R* ^*D*^ */-*) skin samples. Genes are labeled using Ensembl identifiers, as well as associated gene symbols in all cases in which they could be retrieved from Ensembl databases. Differential expression analyses were performed using DESeq2 package (see text for details). A threshold of FDR<0.1 was used to evaluate significant expression changes distinguishing skin samples associated with the two compared phenotypes.
(XLSX)
