# Supplementary figures and images for: Dominant Red Coat Color in Holstein Cattle Is Associated with a Missense Mutation in the Coatomer Protein Complex, Subunit Alpha (COPA) Gene
Source: PLoS One. 2015 Jun 4;10(6):e0128969. doi: 10.1371/journal.pone.0128969 (PMC4456281; doi:10.1371/journal.pone.0128969)

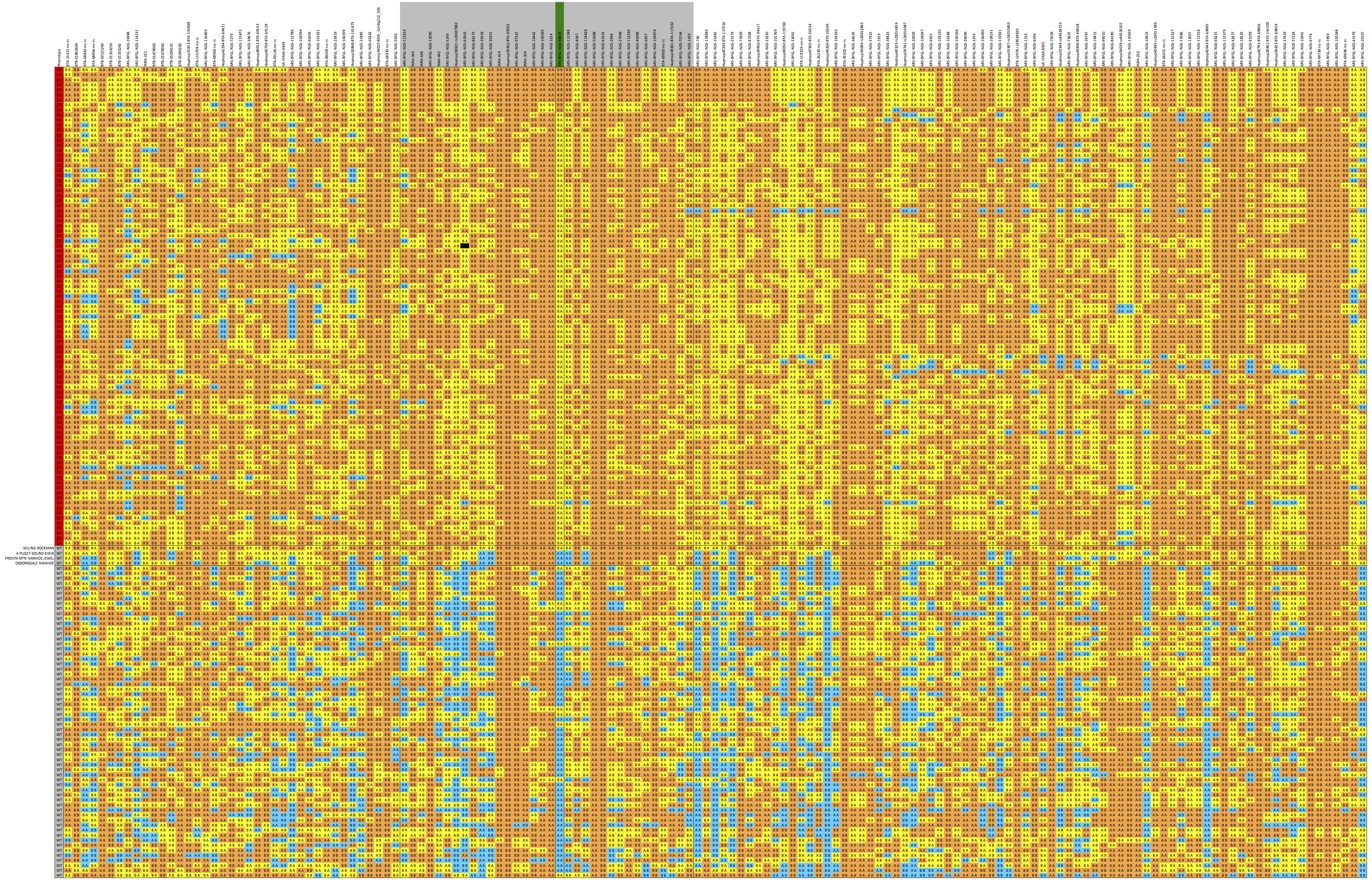

Supplement: S1 Fig — A 2.6 Mb IBD haplotype in Dominant Red animals is located on BTA3 from ARS-BFGL-NGS-112616 @ 7,906,099 bp to ARS-BFGL-NGS-1429 @ 10,462,387 bp. Animals are in rows and SNPs are in columns. Phenotype is indicated in the first column with Dominant Red animals shaded in red and wild-type animals shaded in gray. SNPs are shown that span the entire 10.7 Mb region identified in the linkage analysis. Orange shading indicates homozygotes for the major allele, yellow shading indicates heterozygotes and blue shading indicates homozygotes for the minor allele. The IBD haplotype is shaded in gray and the SNP with the highest GWAS significance shaded in green (ARS-BFGL-NGS-94819 @ 9,722,400 bp). The four ancestors of the founder animal ROSABEL are indicated at the left of the figure. SEILING ROCKMAN shows a haplotype concordant with the major allele from Dominant Red animals extending beyond the 2.6 Mb IBD haplotype. (TIFF) [file pone.0128969.s001.tiff]

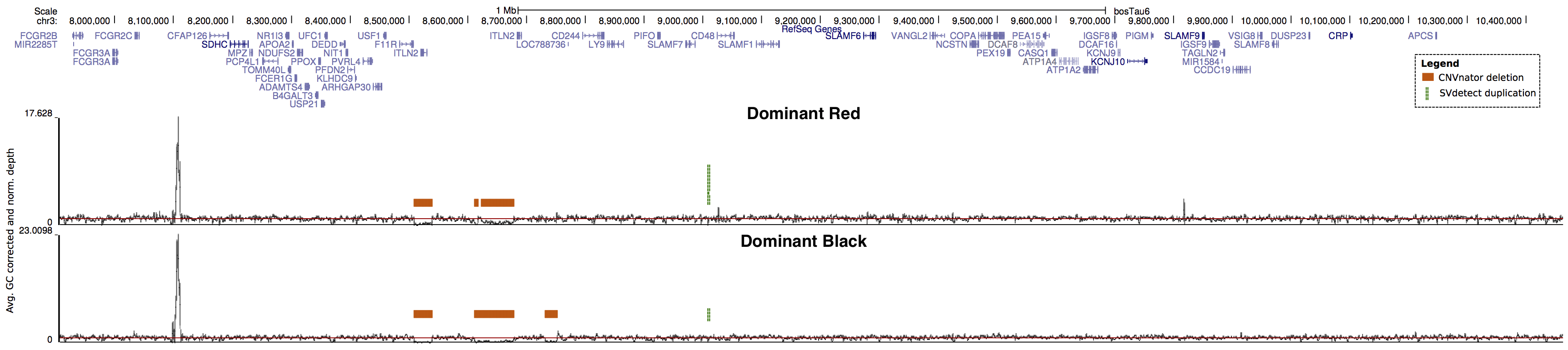

Supplement: S2 Fig — The IBD region was subject to analysis of candidate structural variations using whole genome sequencing reads from a Dominant Red (HOCANF9845383, BROEDERDALE MALTBY CAMILLA) and reference individual (HOCANM10705608, BRAEDALE GOLDWYN). The top track represents RefSeq Gene annotation. Normalized and GC corrected read depth (RD) for the two individuals are found in the bottom two tracks with the mean RD marked with a red line. Significant deletions from CNVnator (orange) and duplications from SVDetect (green) within each individual are overlaid on the RD graph. No other significant structural variations were detected, including analysis with CNV-seq to compare RD between individuals. (TIFF) [file pone.0128969.s002.tiff]
